# Single-Particle Crushing Test of Coated Calcareous Sand Based on MICP

**DOI:** 10.3390/ma17194690

**Published:** 2024-09-24

**Authors:** Shuyue Zhu, Linxian Gong, Zhazha Hu, Yan Xu, Yuanyuan He, Yunyi Long

**Affiliations:** 1 Institute of Resource and Environment, Henan Polytechnic University, Jiaozuo 454000, China; 212203010021@home.hpu.edu.cn (S.Z.); longyunyi@hnlgdx1.wecom.work (Y.L.); 2 Institute of Energy Science and Engineering, Henan Polytechnic University, Jiaozuo 454000, China; zhazha.hu@hpu.edu.cn; 3 Construction Engineering College, Jilin University, Xin Min Zhu Street, Changchun 130026, China; xuyan8102@jlu.edu.cn; 4 Key Laboratory of Geological Hazards on Three Gorges Reservoir Area, China Three Gorges University, Ministry of Education, Yichang 443002, China; heyuanyuan@ctgu.edu.cn

**Keywords:** MICP, coating calcareous sand particle, coating thickness, single particle crushing, particle crushing pattern

## Abstract

Calcareous sand is a crucial construction material for island and reef development and reinforcing it using Microbially Induced Calcite Precipitation (MICP) technology is a promising new method. This study employed 3D scanning technology to assess changes in the particle size and morphology of MICP-treated, coated calcareous sand particles. Single-particle crushing tests were conducted to analyze their crushing strength, crushing energy, crushing modes, and fragment fractal dimensions. The results indicated that MICP treatment significantly increased particle size, surface area, and volume, while reducing flatness. At a cementation solution concentration of 1 mol/L, both crushing strength and crushing energy were optimized. The coated particles exhibited three crushing modes: explosive crushing, mixed crushing, and splitting crushing. Thicker coatings led to a tendency for particles to break into larger fragments through the mixed and splitting crushing modes. Fractal analysis revealed that coating thickness directly affects the local crushing characteristics of the particles.

## 1. Introduction

Calcareous sand, the most common coral reef geotechnical material used in the construction of island and reef infrastructure, has significant engineering implications for island construction and marine engineering. Composed of biological deposits and marine organism skeletal remains, its primary component is calcium carbonate, which has a lower hardness than quartz [1]. It is widely distributed in shallow seas, warm seas, and continental shelf areas. Calcareous sand has a complex particle morphology, significant intra-particle porosity, low strength, and is prone to crushing [2,3]. These unfavorable mechanical properties cause calcareous sand foundations to easily undergo excessive deformation due to particle crushing and slippage under certain loads [4].

Currently, various preventive measures have been taken to address the issue of calcareous sand’s propensity for crushing in island reef engineering construction, mainly including mechanical compaction and chemical reinforcement methods. Mechanical compaction involves compacting calcareous sand using heavy hammers or vibrating equipment to improve its density and bearing capacity. This method, while enhancing the foundation bearing capacity with minimal environmental impact, is convenient to use and widely applied globally [5,6]. However, due to the crushable nature of calcareous sand, mechanical compaction may damage the soil structure and reduce its compressive strength. Additionally, the high porosity of calcareous sand results in suboptimal compaction effects and poor stability. Chemical reinforcement primarily uses cement [7,8], water glass [9], and chemical adhesives [10] to enhance the deformation resistance and bearing capacity of calcareous sand foundations [11]. However, chemical reinforcement methods are typically high-polluting, with these reinforcement materials negatively impacting the environment during production and use. Consequently, conventional mechanical compaction and chemical reinforcement methods for treating calcareous sand struggle to meet the requirements of effectiveness, ecology, and environmental protection, simultaneously. Therefore, there is an urgent need to develop innovative reinforcement methods to improve the mechanical properties of calcareous sand.

In recent years, Microbially Induced Calcite Precipitation (MICP) has gained prominence in soil stabilization applications, involving the repair of concrete cracks [12] and the enhancement of soil mechanical properties [13,14], liquefaction resistance [15,16], erosion resistance [17,18], and heavy metal immobilization [19]. During metabolism, microorganisms produce urease, which hydrolyzes urea to generate ${\mathrm{CO}}_{3}^{2-}$. This reacts with Ca^2+^ to form CaCO_3_ within the soil, which acts as a binding agent [20,21]:

(1) $${\mathrm{CH}}_{4}N_{2}O+4H_{2}O\overset{\mathrm{Ureae}}{\to }H_{2}{\mathrm{CO}}_{3}+2{\mathrm{NH}}_{4}^{+}+2{\mathrm{OH}}^{-}$$

(2) $${\mathrm{HCO}}_{3}^{-}+{\mathrm{OH}}^{-}\to {\mathrm{CO}}_{3}^{2-}+H_{2}O\;$$

(3) $${\mathrm{Ca}}^{2+}+{\mathrm{CO}}_{3}^{2-}\to {\mathrm{CaCO}}_{3}\;\;\;\;\;$$

The surface of microbial cells carries multiple negatively charged functional groups, which can effectively adsorb cations such as Ca^2+^ and Mg^2+^ [22]. These groups act as nucleation sites for CaCO_3_, forming larger crystals and thereby increasing the cementation efficiency of CaCO_3_ [23]. Additionally, urease-producing bacteria create an alkaline environment when hydrolyzing urea, which promotes further growth of calcite. In a higher pH environment (pH 8–9), urease-producing bacteria exhibit higher activity and a stronger urea hydrolysis ability, facilitating calcite precipitation.

Using the MICP method to enhance the bearing capacity of calcareous sand foundations has many advantages, including being environmentally friendly, low-carbon, rapid, efficient, and highly controllable. Moreover, due to the high porosity within single particles of calcareous sand, bacteria can penetrate into the sand, making it possible for MICP to improve the strength of calcareous sand particles. Current research primarily focuses on the compressive strength, liquefaction resistance [24], and permeability [25] of MICP-reinforced calcareous sand at the laboratory unit scale. There is still limited research on the impact of MICP on the crushing strength of individual calcareous sand particles. Considering the rough surface and high internal porosity of calcareous sand particles, the cementation generated by MICP can form a coating layer on the particle surface or fill the internal pores, helping to enhance the particle crushing strength and improve the mechanical properties of calcareous sand particles. Therefore, studying the effect of MICP on improving the crushing strength of calcareous sand particles at the particle scale is particularly important and will help to better reveal the solidification mechanism of MICP-treated calcareous sand.

The CaCO_3_ generated by MICP can exist in calcareous sand in various forms such as cementation, bridging, pore filling, and coating [26,27,28]. Different precipitation modes have significant impacts on the mechanical properties of mineralized calcareous sand particles. The formation of a calcium carbonate coating on the surface of particles plays an important role in enhancing the mechanical properties of the soil. This is because it not only improves the performance of the particle surface but also increases the strength of the particles, indirectly promoting the cementation between particles. This study conducted a series of single-particle crushing tests on coated calcareous sand particles. Additionally, this study is the first to explore the effect of changes in the cementation solution concentration on the coating thickness, as well as the impact of the coating thickness variation on the crushing strength of calcareous sand particles. First, calcareous sand particles with similar shape parameters were selected through 3D scanning. These particles were then reinforced with MICP solutions of different cementation concentrations to prepare the coated calcareous sand particles. Subsequently, single-particle crushing tests were performed using a self-developed particle mechanics testing system. By analyzing the crushing strength, crushing energy, crushing process and modes, and fragment characteristics of the coated calcareous sand particles, this study explores the compressive crushing behavior of MICP-coated calcareous sand particles.

## 2. Materials and Methods

### 2.1. Test Particles and Morphology Extraction

#### 2.1.1. General Chemical and Physical Features

**Figure 1:**
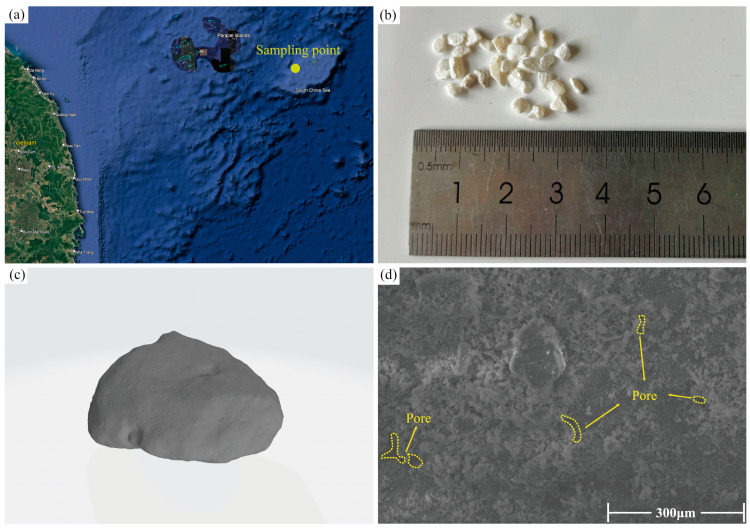
Calcareous sand sampling sites and sand images: (**a**) Sampling site of calcareous sand in South China Sea; (**b**) Calcareous sand image and particle size; (**c**) Three-dimensional scanning image of calcareous sand particle; (**d**) SEM image of 2000× calcareous sand.

The calcareous sand used in this study was sourced from a certain island reef in the Nansha Islands of China (Figure 1a). The sampling area was widely distributed with calcareous sand of various particle sizes, and particles of 3–4 mm were selected for the experiments. As shown in Figure 1b, the particle morphology of the South China Sea calcareous sand is highly irregular, with rough surfaces, sharp edges, and numerous pores. Through 3D scanning, detailed images of the particles were obtained (Figure 1c), revealing the uneven surface of the calcareous sand particles. This unevenness is due to the composition of calcareous sand particles from marine biological skeletons, shells, and other calcareous remains, and their deposition in the marine environment, influenced by dynamic forces such as waves and tides. Under a scanning electron microscope magnified at 2000×, the surface of the particles can be seen to be covered with a large amount of calcium carbonate powder and numerous small pores (Figure 1d).

**Figure 2:**
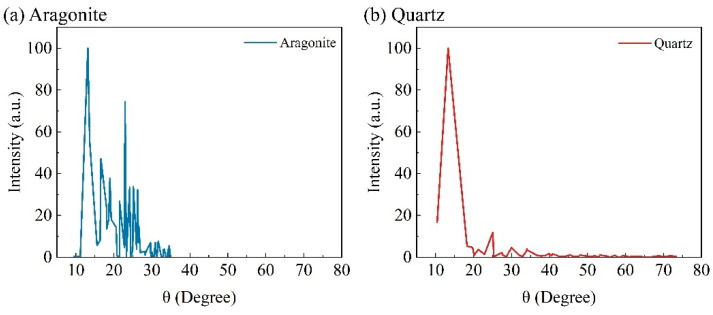
Diffraction peak intensity of main mineralized components of calcareous sand particles.

Figure 2 shows the mineral composition of the calcareous sand particles obtained through X-ray diffraction (XRD) analysis. The primary mineral components of the calcareous sand particles are aragonite (CaCO_3_, unit cell parameters: 4.9598 × 7.9641 × 5.7379, crystal system: orthorhombic) and quartz (SiO_2_, unit cell parameters: 4.9141 × 4.9141 × 5.406, crystal system: hexagonal). Since calcareous sand is a carbonate mineral, it contains a high content of aragonite [29,30]. Additionally, although calcareous sand typically forms in shallow marine environments, other types of sediment sources, such as terrigenous sediments (e.g., quartz particles), can also be present in these areas. Rivers, storms, and other natural forces transport terrigenous sediments into shallow marine zones, where they mix with calcareous sand and co-deposit.

#### 2.1.2. Morphology Extraction Tests

**Figure 3:**
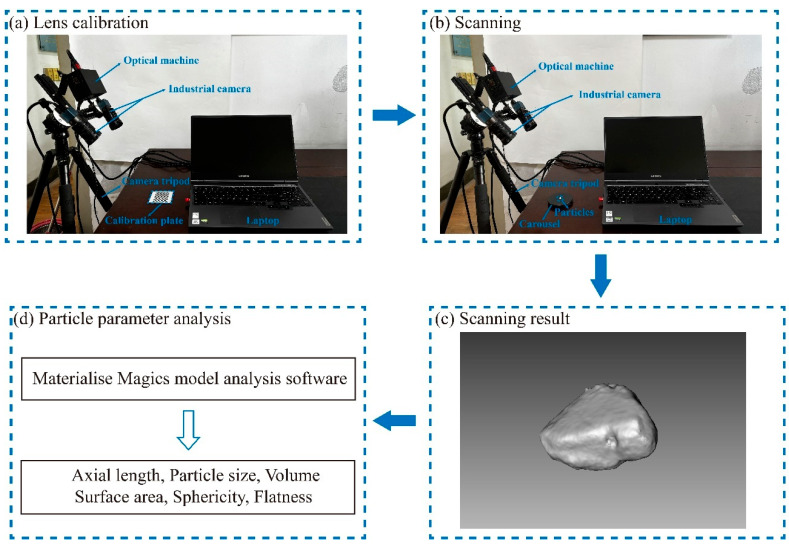
Morphology extraction tests’ process of calcareous sand and coated calcareous sand particles.

In this study, 3D scanning technology was utilized to scan untreated calcareous sand particles as well as those treated with different concentrations of cementation solutions, to comparatively analyze the changes in particle shapes under different treatment conditions. We independently constructed a 3D scanner system, with hardware components including a light machine, industrial camera, computer, and camera tripod, and software FlexScan3D 3.3 (Figure 3). Before starting the scan, it was necessary to precisely adjust the clarity and brightness of the industrial camera and calibrate the distance between the two lenses. Once calibration was completed, the scanner should not be moved. Next, a rotatable specimen stage was placed within the field of view of the lenses, and the particles were placed on the stage for scanning. During the scanning process, the particles needed to be continuously rotated and flipped to ensure that all faces and parts were fully scanned. The software automatically recognized and marked feature points during scanning. After scanning one side, all scanned images were combined, and then the other side of the particle was scanned. Once all sides of the particles were scanned, the images of the front and back sides of the particles were merged. Throughout the process, it was essential to ensure the quality and accuracy of the scanned images. Finally, all scanned images of the particles were combined and exported as STL files. The STL files were then imported into Materialise Magics 27.0 to analyze relevant parameters such as particle size, surface area, and volume.

### 2.2. Bacteria Solution and Cementation Solution

**Figure 4:**
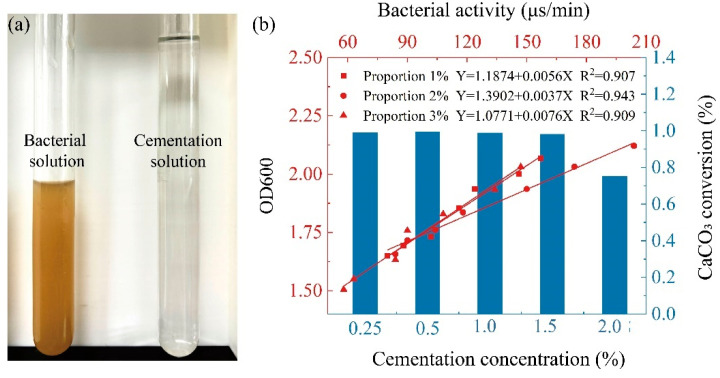
MICP treatment solution used in the test: (**a**) Bacteria solution and cementation solution; (**b**) The bacterial activity at different inoculated ratios and the conversion rate of CaCO_3_ at optimal inoculated ratios.

The MICP solution was composed of bacterial solution (BS) and cementation solution (CS) [31,32] (Figure 4a). In this experiment, the urease-producing microorganism Bacillus pasteurii [21,33,34] (ATCC 11859) was obtained from the Shanghai Microbiological Culture Collection Co., Ltd. (Shanghai, China). This chemoheterotrophic microorganism is a Gram-positive bacterium known for its significant urease production and high urease activity, making it widely used in geotechnical engineering. The cementation solution was a mixture of urea and calcium chloride, which primarily provides abundant ${\mathrm{CO}}_{3}^{2-}$ and Ca^2+^ for the MICP reaction.

**Figure 5:**
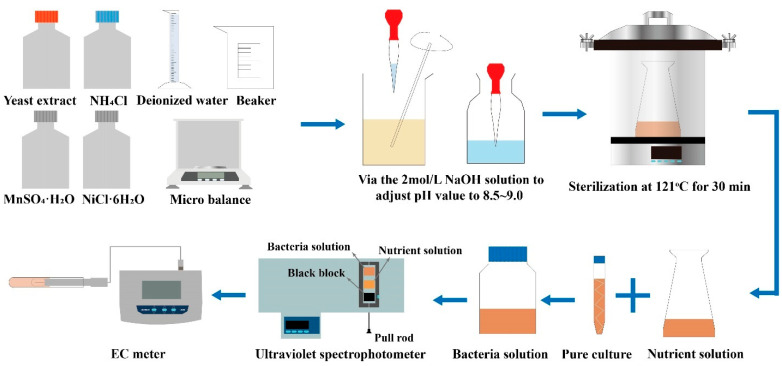
Microbial culture process and test of BS activity and OD600.

In this study, microbial culture was scaled up to obtain sufficient amounts of bacterial solution. Figure 5 illustrates the detailed process for microbial culture and activity measurement. First, the culture medium was prepared according to the composition and quantities listed in Table 1, and the pH was adjusted to between 8.5 and 9.0 using a 2 mol/L NaOH solution. The prepared medium was sterilized at 121 °C under high pressure for 30 min and then cooled to room temperature. In a sterile laminar flow hood, the bacterial strain from the slant culture was inoculated onto solid culture medium and incubated at 30 °C for 24 h. Subsequently, colonies from the solid culture medium were inoculated into 100 mL of liquid culture medium and incubated at 30 °C with shaking at 180 rpm for 24 h. To obtain bacterial solutions with different inoculation ratios, the cultured bacterial solution was inoculated into larger volumes of liquid culture medium at ratios of 1%, 2%, and 3%, and further incubated with shaking at 180 rpm for 48 h. Finally, the optical density at 600 nm (OD600) of the bacterial solutions at different inoculation ratios was measured using a UV spectrophotometer, setting different dilution factors.

According to the method proposed by Whiffin et al. [35], the activity of the bacterial solution was measured, resulting in a relationship between the inoculation ratio, dilution factor, and bacterial solution activity (Figure 4b). The activity of the bacterial solution is determined by measuring the average change in its conductivity. The conductivity value of the bacterial solution reflects its ability to hydrolyze urea. To measure this, the bacterial solution is mixed with urea in a 1:9 ratio in a test tube, and the change in conductivity is recorded over a five-minute period. Finally, the average change in conductivity is calculated. There is a linear relationship between these factors, and the activity of the bacterial solution gradually decreases with increasing dilution. Additionally, 2% was found to be the optimal inoculation ratio, as it achieved the best microbial growth and proliferation conditions. Subsequently, the calcification test determined the mineralization capacity of microorganisms under the optimal inoculation ratio and different cementation concentrations (Figure 4b). It can be seen that when the cementation solution concentration ranged from 0.25 to 1.5 mol/L, the calcium carbonate conversion rate remained relatively high at over 90%. However, at 2 mol/L, the calcium carbonate conversion rate significantly decreased, as the high cementation concentration inhibited the microbial mineralization capacity [36]. From the figure, it can be observed that both the calcium carbonate conversion rate and the amount of calcium carbonate generated reached optimal levels at a cementation concentration of 1.0 mol/L.

### 2.3. MICP Treatment for Individual Particles

**Figure 6:**
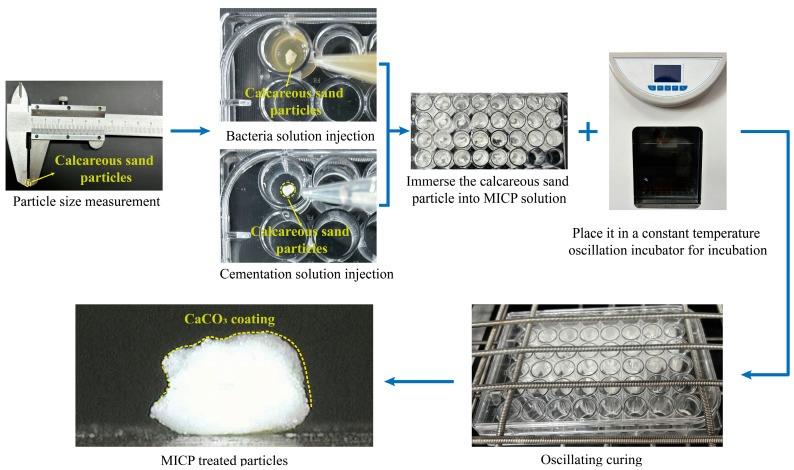
MICP preparation process of coated calcareous sand particles.

Figure 6 shows the MICP preparation process for coated calcareous sand particles. First, particles were precisely measured using a vernier calliper, determining their diameter by measuring the long axis (L), intermediate axis (I), and short axis (S). The particle diameter (d) was calculated using the formula: (d = (L + I + S)/3). The calcareous sand particles used in this study had diameters ranging from 3 to 4 mm, with an average diameter of 3.5 mm determined by (d_50_). To improve the stability of the test results, the initial calcareous sand particles selected for the test were as close to spherical as possible. Subsequently, the particles were placed in a 48-well plate for treatment, with a bacterial solution to cementation solution ratio of 1:4. The bacterial solution was added first to allow microbial colonization, followed by a six-hour curing period before adding the cementation solution and curing for an additional 24 h. This provided sufficient time for the urease-producing bacteria to hydrolyze urea and generate CaCO_3_ precipitates. The study set up control and experimental groups: the control group consisted of untreated calcareous sand particles (α_1_), while the experimental groups included calcareous sand particles treated with 0.5 mol/L (α_2_), 1.0 mol/L (α_3_), and 1.5 mol/L (α_4_) cementation solutions. The effectiveness of the treatments was evaluated by comparing the crushing strength of particles under different treatment conditions. Each group included 30 calcareous sand particles, with the number of particles selected based on existing single-particle crushing test studies on sand particles [37], railway ballast [38], and rockfill materials [39]. McDowell [40] pointed out that the deviation between the strength determined from 30 tests and the true strength can be controlled within 25% at a 95% confidence interval. Therefore, conducting 30 tests provides relatively stable single-particle strength results for both calcareous sand and coated calcareous sand.

### 2.4. Single-Particle Crushing Tests

**Figure 7:**
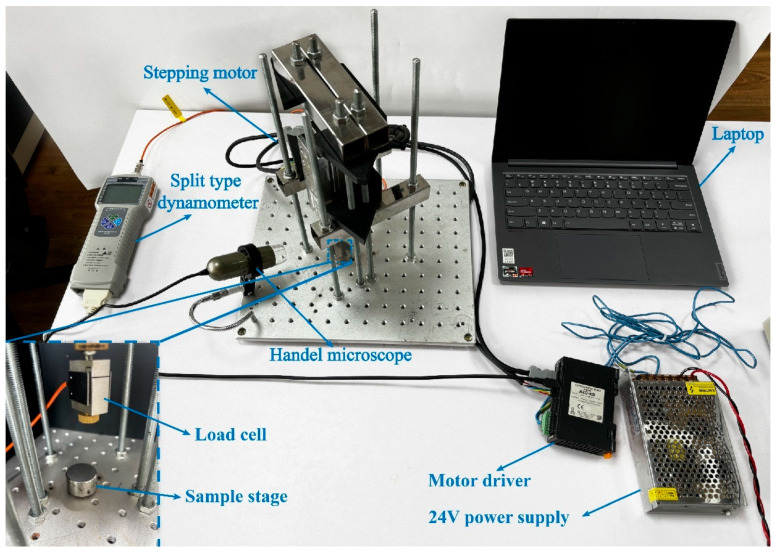
Particle mechanics test system.

In this study, a self-developed particle mechanics testing device was used to measure the mechanical properties of calcareous sand and coated calcareous sand particles (Figure 7). The device consists of a stepper motor, driver, force sensor, handheld microscope, external power supply, computer, and sample stage. It is capable of testing both the compressive and tensile strength of particles. In the experiments, single-particle crushing tests were conducted on both the experimental group and the control group particles. The displacement distance and speed of the motor shaft can be precisely controlled using the MEXE02 Ver.4 software. The stepper motor applied vertical pressure at a speed of 0.5 mm/s until the particles fractured, with the force and displacement during the process measured by the force sensor. Additionally, the handheld microscope captured high-resolution images of the single-particle crushing process, allowing for a detailed evaluation of the crushing behavior of calcareous sand and coated calcareous sand particles on a microscale.

### 2.5. Scanning Electron Microscopy (SEM) Tests

In this study, the microstructure of calcareous sand particles before and after MICP treatment was observed using a Quanta FEG 250 scanning electron microscope (SEM) (FEI, Hillsboro, OR, USA). SEM analysis helps to investigate the micro-morphology and structure of both untreated and coated calcareous sand samples. Before the experiment, the samples were sputter-coated with gold and their bases were treated with conductive adhesive to enhance conductivity. Subsequently, these gold-coated samples were placed in the sample chamber of the scanning electron microscope for observation, and high-resolution micrographs were obtained.

## 3. Results and Discussion

### 3.1. Morphologies of Coated Calcareous Sand

**Figure 8:**
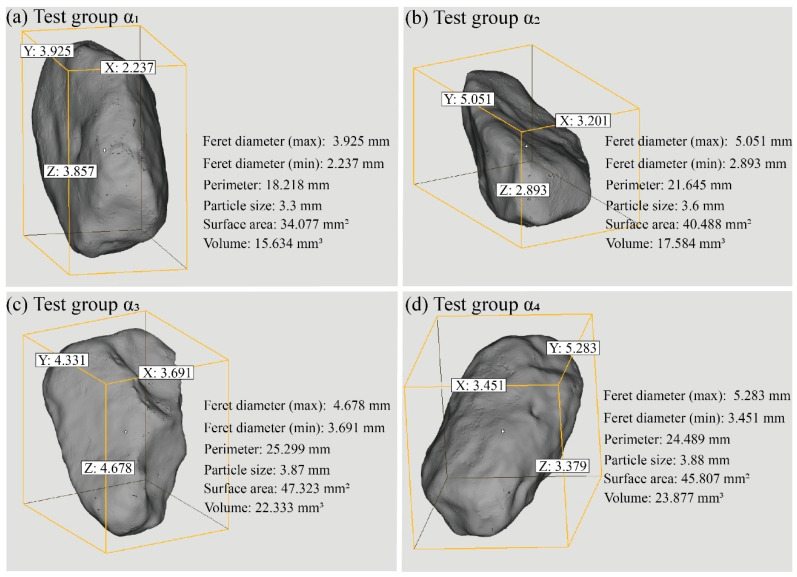
The shape and related parameters of scanned particles.

The shape of calcareous sand particles has a direct impact on the mechanical properties of MICP-treated, coated calcareous sand. It is important to note that before MICP treatment, we measured and quantified the shape parameters of calcareous sand particles through 3D scanning tests, and selected particles with similar shape parameters as raw materials to minimize the influence of particle shape on the experimental results. Currently, research on particle shape quantification is mainly based on two-dimensional sections, which cannot accurately reflect three-dimensional characteristics such as sphericity. Therefore, this study used a 3D scanner to obtain three-dimensional images of calcareous sand particles before and after MICP treatment and imported them into the Materialise Magics software to analyze parameters such as particle size, surface area, and volume (Figure 8). Some of these parameters need to be calculated based on the basic characteristics and information of the particles using specific formulas [3]. Additionally, by measuring the lengths of the x, y, and z axes of the particles, the long axis, intermediate axis, and short axis of the calcareous sand particles can be clearly distinguished. The flatness (E) and sphericity (S) of the coated calcareous sand particles are calculated using the following formulas:

E = L/B(4)

S = R_i_/R_c_ (5)

where (L) is the Feret diameter (max), (B) is the Feret diameter (min), (R_i_) is the inscribed circle radius of the particles (max), (R_c_) is the external radius of the particles (min), (E) is the flatness of the particles, and (S) is the sphericity of the particles.

Figure 8 shows the basic size parameters of the control group calcareous sand particles (α_1_) and the experimental groups (α_2_, α_3_, α_4_) coated with MICP, as obtained from the 3D scanning tests. The lengths of the long axis, intermediate axis, and short axis of the untreated calcareous sand particles in the α1 group are all lower than those of the MICP-treated particles in the experimental groups. Compared to the control group, the particle size and perimeter of the MICP-treated, coated calcareous sand particles increased to some extent, and this increase was more pronounced with higher cementation solution concentrations. Additionally, the volume and surface area of the coated calcareous sand particles also increased. The generation of calcium carbonate crystals by microbial mineralization forms a coating of a certain thickness on the particle surface, which is the reason for the increase in the basic size parameters of the particles.

**Figure 9:**
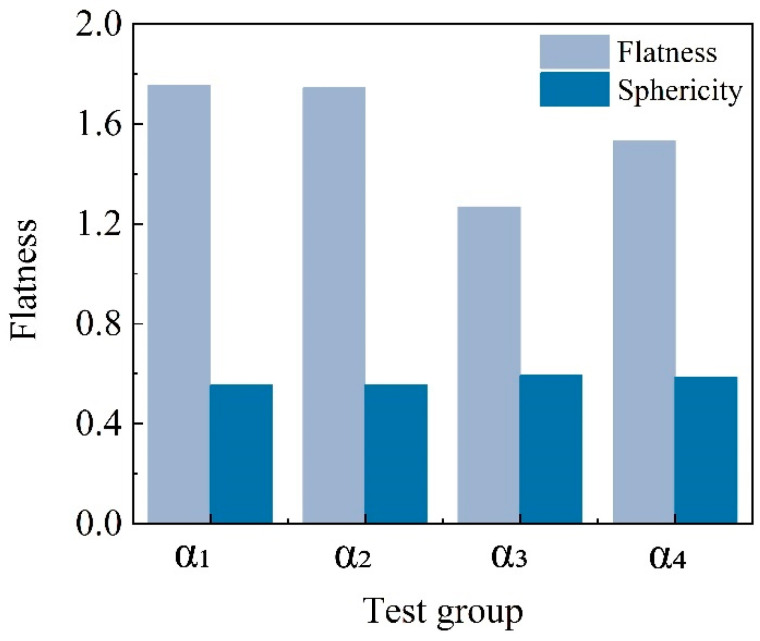
Sphericity and flatness of particles under different treatment methods.

Figure 9 shows the flatness and sphericity parameters of the control group calcareous sand particles (α_1_) and the experimental groups (α_2_, α_3_, α_4_) coated with MICP. The untreated calcareous sand particles in the control group exhibit higher flatness, with an overall flatter shape that primarily extends along the *x*-axis and *y*-axis directions. In contrast, the MICP-treated particles in the experimental groups have a noticeable coating layer formed by calcium carbonate generated through microbial mineralization, which increases the length of the particles along the *z*-axis and thereby reduces their flatness. Moreover, there is an inverse relationship between the sphericity and flatness of the particles. A higher sphericity index indicates that the particles are closer to being spherical. Microbial mineralization has somewhat improved the sphericity of the particles, but due to the inherent particle size characteristics of calcareous sand, the change in the sphericity index is limited. Although there is an increase, it remains at a relatively low level.

### 3.2. Microscopic Structure of Coated Calcareous Sand

**Figure 10:**
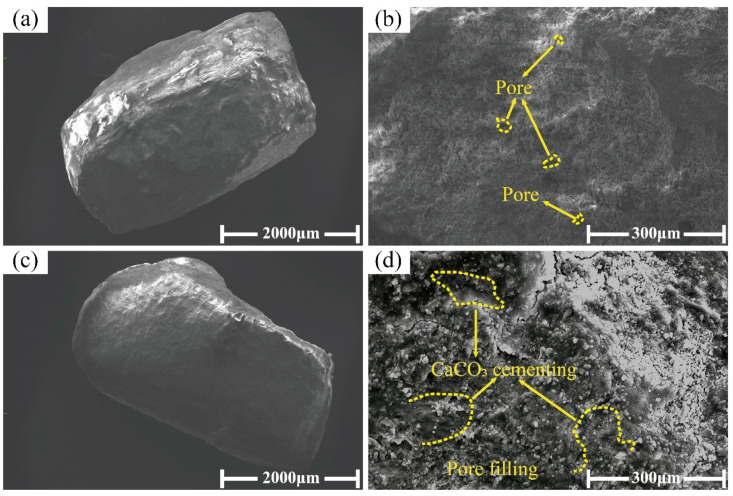
SEM images of calcareous sand particles: (**a**) SEM image of 60× particles without MICP treating; (**b**) Zoomed in at 2000× without MICP treating; (**c**) SEM image of 60× particles under MICP treating; (**d**) Zoomed in at 2000× under MICP treating.

The structural changes in the calcareous sand after MICP treatment are the fundamental reason for the enhancement of its crushing strength. Therefore, we used a scanning electron microscope (SEM) to observe the microstructure of the calcareous sand and coated calcareous sand particles. Figure 10a,c show the micrographs of the untreated and MICP-treated calcareous sand particles at 60× magnification. It can be observed that the surface of the untreated calcareous sand particles is uneven and rough (Figure 10a). However, after MICP treatment, the surface of the coated calcareous sand particles is relatively smooth, with the unevenness significantly reduced (Figure 10c). Figure 10b,d show the micrographs of the calcareous sand and coated calcareous sand particles at 2000× magnification. At this higher magnification, the untreated calcareous sand particles exhibit dense and tiny pores on the surface, with a large amount of fine and loose calcareous sand powder adhered to it (Figure 10b). In contrast, the surface of the MICP-treated coated calcareous sand particles is covered with a calcium carbonate coating layer, and the original pores on the particle surface are filled with calcium carbonate (Figure 10d). This calcium carbonate coating forms a layer with a certain compressive strength on the particle surface, effectively increasing the crushing strength of the calcareous sand particles and enhancing their mechanical properties.

### 3.3. The Impact of Coating Thickness on Crushing Behavior

#### 3.3.1. Residual Probability and Characteristic Strength

For the calculation of the single-particle crushing strength, the widely adopted method used is σ = F/d^2^, where σ represents the compressive stress experienced by the material, F is the measured force during the test, and d is the particle diameter [41,42]. Generally, the crushing strength of brittle granular materials exhibits significant variability. The Weibull distribution [43] has been widely used to study the brittle fracture of solid materials and has been proven applicable for researching the crushing of brittle geotechnical materials such as calcareous sand and quartz sand [37,42]. The mathematical expression of the Weibull distribution is:

(6) $$\mathrm{Ps}\left(d\right)=\exp\left[{-\left(\frac{\sigma_{c}}{\sigma_{c0,d}}\right)}^{m}\right]$$

In the formula, d is the particle diameter; Ps(d) is the survival probability of the particles, which is the proportion of particles that remain unbroken at a certain stress level relative to the total number of particles. The value σ_c_ is the crushing strength of the particles, calculated by σ_c_ = F_f_/d^2^, where F_f_ is the peak pressure. And σ_c0,d_ is the characteristic strength of particles with diameter (d), which represents the strength at which 37% of the particles do not fracture. This value is used to characterize the strength of particles of a certain diameter or under a specific treatment method.

**Figure 11:**
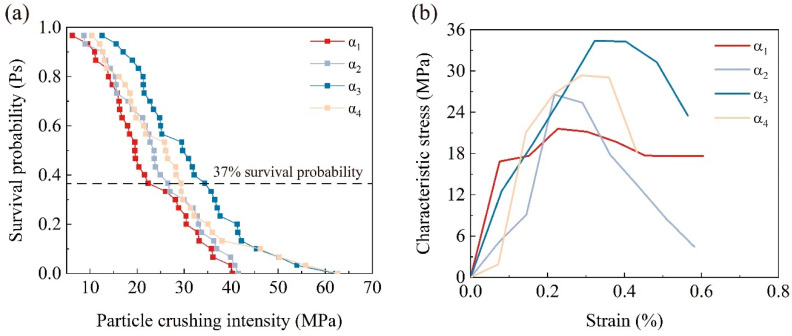
The experimental data of calcareous sand and coated calcareous sand particles: (**a**) Survival probability of calcareous sand and coated calcareous sand particles; (**b**) Characteristic stress of calcareous sand and coated calcareous sand particles.

Figure 11 shows the survival probability and characteristic strength of particles treated with different concentrations of the cementation solution. The survival probabilities of calcareous sand particles under different treatment methods exhibit a roughly similar declining trend, reflecting the uniformity and accuracy of the tests (Figure 11a). However, when the cementation solution concentration is 1 mol/L and 1.5 mol/L, the crushing strength of the particles increases at the same survival probability, leading to an increase in the characteristic strength of the particles. Figure 11b shows that the characteristic strength of calcareous sand particles is enhanced to some extent after MICP treatment. However, with the increase in the cementation solution concentration, the characteristic strength of the calcareous sand initially increases and then decreases, reaching a maximum of 34.402 MPa at a concentration of 1.0 mol/L. Subsequently, when the concentration increases to 1.5 mol/L, a decreasing trend is observed. This phenomenon can be attributed to the fact that a 1.0 mol/L cementation solution enables microorganisms to have a stronger mineralization capacity, generating more calcium carbonate compared to a 0.5 mol/L cementation solution, with the calcium carbonate crystals primarily forming blocky shapes. In contrast, at a cementation solution concentration of 0.5 mol/L, the calcium carbonate crystals are mainly spherical [44]. However, when the cementation solution concentration is increased to 1.5 mol/L, the excessively high concentration inhibits microbial activity, negatively impacting the effectiveness of the MICP treatment [45].

**Figure 12:**
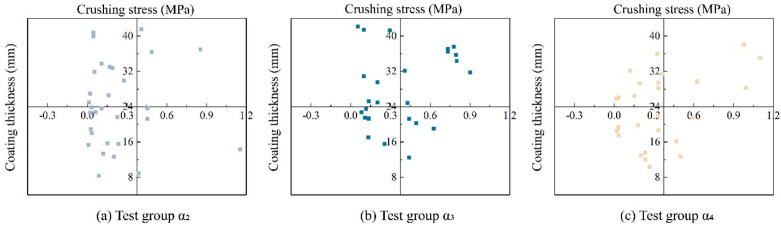
Relationship between coating thickness and particle strength.

Figure 12 shows the distribution of the crushing strength of 30 particles in different experimental groups, corresponding to the coating thickness. In Figure 12a, it can be seen that for the coated calcareous sand particles treated with a 0.5 mol/L cementation solution, the coating thickness values are generally low, ranging between 0 and 0.3 mm. At this stage, the crushing strength values of the calcareous sand are relatively low, between 8 and 24 MPa. However, when the cementation solution concentration is increased to 1 mol/L, there is a significant increase in the coating thickness, with nearly half of the particles having coating thickness values at a higher level, between 0 and 0.9 mm. At this concentration, the crushing strength values of the calcareous sand are mostly within the range of 24 to 44 MPa for more than 50% of the particles. As the cementation solution concentration is further increased to 1.5 mol/L, microbial mineralization is inhibited, resulting in less calcium carbonate being produced. Consequently, the coating thickness on the particle surfaces is somewhat reduced compared to the particles treated with a 1 mol/L cementation solution, primarily ranging between 0 and 0.3 mm. Simultaneously, the crushing strength of the particles also decreases, with about 50% of the coated calcareous sand particles having crushing strengths between 8 and 24 MPa, and another 50% having crushing strengths between 24 and 40 MPa. This phenomenon can be attributed to the limited amount of calcium carbonate produced at lower cementation solution concentrations, leading to thinner coating layers on the particle surfaces and consequently lower crushing strengths. With the increase in the cementation solution concentration, more calcium carbonate cementation is produced through microbial mineralization, forming thicker coating layers and thereby enhancing the crushing strength of the coated calcareous sand particles. Additionally, some particles exhibit an extremely high crushing strength (≥40 MPa), which contributes to the higher characteristic strength within this treatment group (Figure 12b). However, excessively high cementation solution concentrations lead to a more concentrated range of particle crushing strengths (Figure 12c), with fewer instances of an extremely high crushing strength. This indicates that while the MICP treatment effect is inhibited, more than half of the particles still maintain relatively high crushing strengths, resulting in a higher characteristic strength (higher than that of the α_2_ group).

#### 3.3.2. The Dispersion of Particle Strength Distribution

The Weibull modulus (m) is an indicator used to measure the dispersion of the strength distribution of coated calcareous sand particles. The larger the m value, the smaller the dispersion of particle strength. To facilitate the calculation of m and σ_c0, d_, we transform Equation (6) into Equation (7):

(7) $$\ln\left\{\ln\frac{1}{\mathrm{Ps}(d)}\right\}=m\left(\ln\sigma_{c}-\ln\sigma_{c0,d}\right)$$

**Figure 13:**
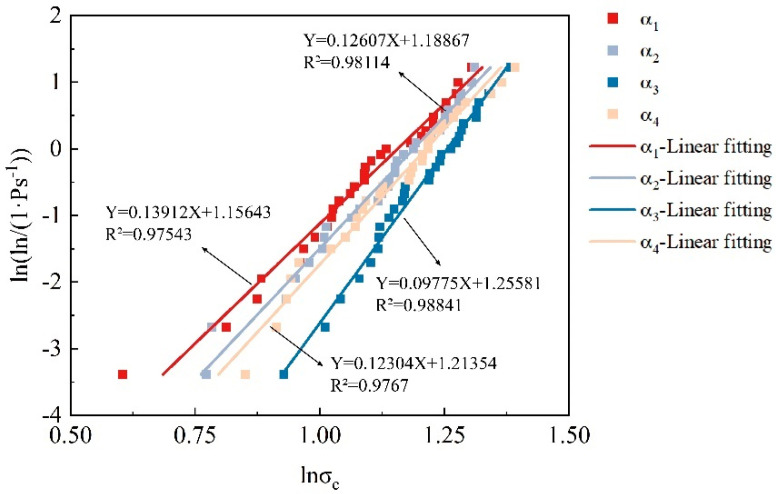
Weibull modulus of calcareous sand treated with different CS concentration.

Using Equation (7), we plotted the Weibull distribution graphs of the strength of coated calcareous sand particles under different treatment methods (Figure 13). The results show that the crushing strength of calcareous sand particles treated with different concentrations of the cementation solution can be well described by the Weibull distribution, with a high degree of fit. In Figure 13, the slope of the fitted lines corresponds to the Weibull modulus m of the coated calcareous sand particles treated with different concentrations of the cementation solution. When ln{ln[1/Ps(d)]} = 0, the particle crushing strength σ_c_ corresponds to the characteristic strength σ_c0, d_ of the coated calcareous sand particles at that coating thickness. Additionally, after MICP treatment, a coating of a certain thickness is formed on the particle surfaces, and the coating thickness increases with the concentration of the cementation solution.

Table 2 shows the coating thickness, characteristic strength, and Weibull modulus of the particles in the control and experimental groups. It can be seen that there is a certain relationship between the concentration of the cementation solution, coating thickness, Weibull modulus, and characteristic strength. After MICP treatment, a calcium carbonate coating of a certain thickness forms on the particle surfaces. As the cementation solution concentration increases, the coating thickness first increases and then decreases, reaching a peak at a concentration of 1 mol/L. However, when the concentration reaches 1.5 mol/L, the excessively high concentration inhibits the microbial mineralization ability, leading to a reduced increase in coating thickness. The variation in coating thickness directly affects the Weibull modulus and the characteristic strength of the particles. An increase in coating thickness generally enhances the characteristic strength of the particles. As the coating thickness increases, the dispersion in particle crushing strength also increases. This may be because, at the optimal cementation solution concentration, microbial mineralization significantly enhances the crushing strength of some particles. However, when microbial mineralization weakens, the distribution of the particle crushing strength values becomes more concentrated, reducing their dispersion. In summary, the concentration of the cementation solution has a complex and interrelated effect on the coating thickness, Weibull modulus, and characteristic strength. An appropriate cementation solution concentration helps optimize the mechanical properties of the particles.

#### 3.3.3. Single-Particle Crushing Energy

**Figure 14:**
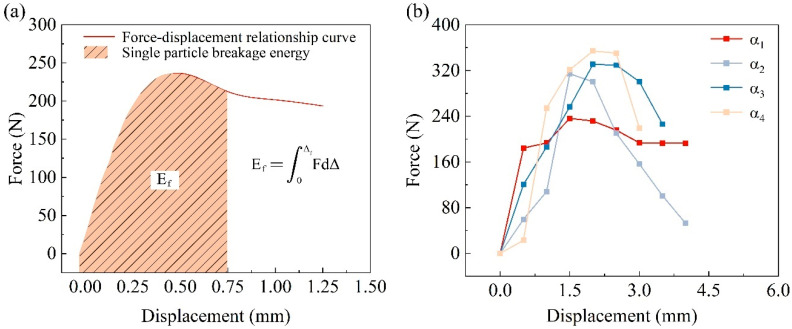
Calcareous sand single-particle crushing energy: (**a**) Single-particle crushing energy calculation method; (**b**) Fracture force-displacement image of calcareous sand particles.

The single-particle crushing test can directly measure the maximum peak force and the corresponding displacement that a single particle withstands at the moment of crushing, thereby calculating the crushing strength of the individual particle. However, the crushing energy of a single particle cannot be directly obtained through the experiment. Existing research indicates [46] that the crushing energy of a single particle can be described by an integral function, which is the integral of the crushing force F with respect to the displacement Δ (Figure 14), as shown in the following equation:

(8) $$E_{f}=\int_{0}^{\Delta_{f}}\mathrm{Fd}\Delta$$

In the equation, E_f_ represents the single-particle crushing energy, F is the crushing force, and Δ is the displacement. For single-particle crushing energy, even though particles of the same material may have similar diameters and shapes, their crushing energy can still differ. This study statistically analyzed the average single-particle crushing energy under the same particle diameter conditions (Table 3) [47]. The crushing energy of calcareous sand particles increases with the coating thickness and the peak crushing force. This is mainly because the calcium carbonate cementation layer formed on the particle surface after the Microbially Induced Calcite Precipitation (MICP) treatment increases the coating thickness, thereby enhancing the particle’s peak crushing force. When the applied load exceeds the load-bearing capacity of the particle, the particle breaks, releasing more energy accordingly. Although the 1.5 mol/L cementation solution concentration inhibits the effect of MICP, reducing the rate of increase in coating thickness, the particles treated with this concentration had a larger initial diameter (3.473 mm), and after the coating increase, their diameter reached 3.667 mm, which is larger than the particles treated with a 1 mol/L cementation solution (3.533 mm). Therefore, their peak crushing force increases, leading to a corresponding increase in crushing energy.

### 3.4. Crushing Process and Pattern

**Figure 15:**
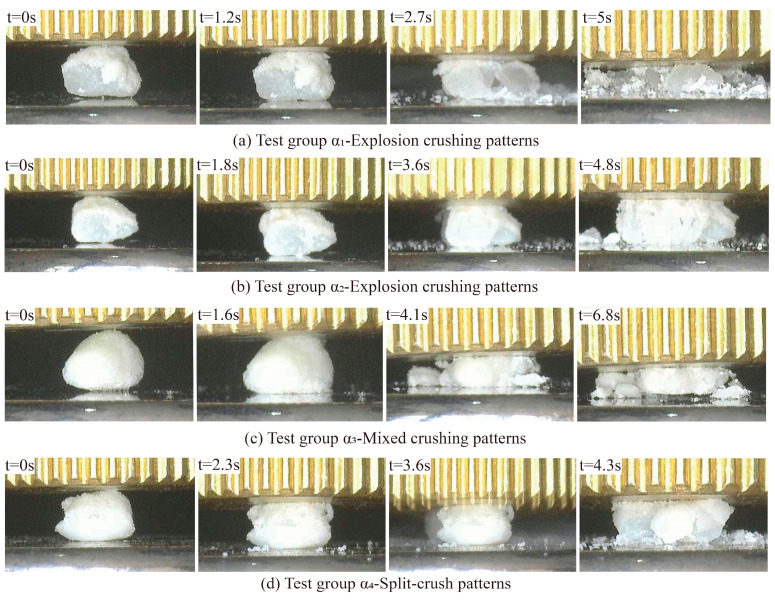
Evolution of particle fragmentation in different experimental groups.

Figure 15 illustrates the evolution of the shape and size of calcareous sand particles during the compression process over time. Before the experiment, the loading plate lightly touches the particle surface to ensure that no force is applied, and the particles retain their original shape. As compression begins, the particles undergo deformation at the first time point, primarily due to the compression of internal pore spaces, indicating that the particles are in the elastic deformation stage. With the continued displacement of the loading plate, the particles quickly reach peak stress at the subsequent time points and then fracture; in some images, particle splashing can be observed. Given the small volume and limited strain range of calcareous sand particles, they complete the crushing process within a short time and with a small displacement of the loading plate. The final time point shows the particles crushed into fragments of varying sizes.

**Figure 16:**
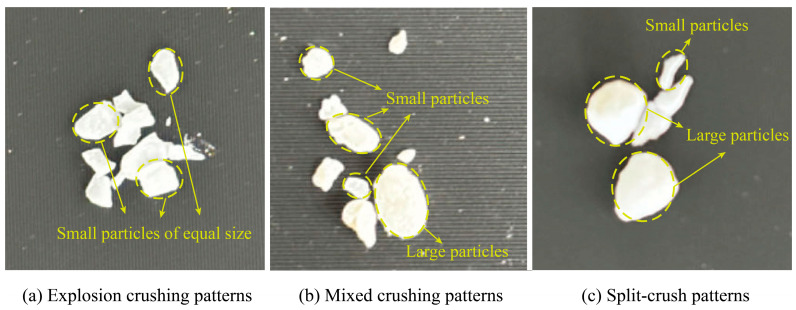
The planform of coating calcareous sand particle crushing patterns under treatment of different CS concentrations.

The particles in the control group and different experimental groups exhibited various crushing modes (Figure 15). The single-particle crushing modes of calcareous sand and coated calcareous sand primarily include explosive crushing (Figure 15a,b), mixed crushing (Figure 15c), and splitting crushing (Figure 15d). The fragments of coated calcareous sand particles broken under different modes show significant differences. In the explosive crushing mode, the particles are shattered into many uniformly small particles; in the mixed crushing mode, the particles break into one to two larger fragments and several smaller ones; while in the splitting crushing mode, the particles primarily fracture into two to three larger fragments with fewer small particles. To more clearly demonstrate the changes in particle crushing modes after treatment with different concentrations of the cementation solution, Figure 16 presents the top views of the fragments from the experimental groups. There is a transition from explosive crushing to splitting crushing. This change can be attributed to the effective filling of internal micropores in the calcareous sand by calcium carbonate produced through microbial mineralization, which enhances the overall crushing strength, causing the particles to tend to break into larger fragments rather than fine particles under stress. Additionally, the change in crushing mode is closely related to the thickness of the calcium carbonate coating. The coating wraps around the calcareous sand particles, providing a certain degree of connectivity, which makes the particles less likely to shatter into many small fragments upon forceful crushing. The increase in coating thickness strengthens this effect, resulting in a higher proportion of larger fragments post-crushing. These findings further confirm the effectiveness of the MICP treatment in enhancing the mechanical properties of calcareous sand.

### 3.5. Fragment Characteristics of Coated Calcareous Sand after Crushing

Fractal theory was initially used to describe the self-similarity between the local and overall properties of an object, originating from the measurement of the British coastline [48]. Numerous studies by F. Yu [49,50] and others have shown that natural geomaterials also follow a fractal distribution when they break into smaller particles. For single-particle crushing, the fractal characteristics can be expressed by the relationship between the number of newly generated sub-particles N(θ > d) and the particle diameter (d), as shown in the following equation [51]:

N(θ > d) = C/d^D^ (9)

where N(θ > d) is the number of particles with a diameter θ > d; C is a constant; D is the fractal dimension, with a larger fractal dimension indicating that the particles have broken into more fine particles. For fractal granular materials, the fractal dimension is related to the Weibull modulus by the following relationship [52]:

D = 3 m/1 + m(10)

By substituting the Weibull modulus of the crushing strength of coated calcareous sand particles into Equation (8), the fractal dimensions D of the particle local crushing state for the different treatment groups (α_1_), (α_2_), (α_3_), and (α_4_) were calculated as 0.366, 0.336, 0.267, and 0.329, respectively. The results indicate that the fractal degree of particle local crushing is related to the coating thickness. As shown in Table 4, the fractal dimension decreases with the increase in coating thickness, suggesting that as the concentration of the cementation solution increases, the coating thickness increases, leading to a decrease in the fractal degree of local crushing. Furthermore, there is a relationship between the fractal dimension and the coating thickness. This is related to the formation of a coating of certain thickness on the particle surface after MICP treatment. The trend of an increase in coating thickness with the change in the cementation solution concentration follows a pattern of initially increasing and then decreasing.

## 4. Conclusions

This study focused on calcareous sand particles coated through MICP mineralization and it conducted single-particle crushing tests on particles with identical shapes to investigate their crushing behavior, yielding the following conclusions:

1. The basic size parameters of MICP-treated coated calcareous sand particles were precisely quantified using 3D scanning technology. The results showed that the MICP treatment significantly altered the surface structure of calcareous sand particles, leading to increases in particle size, surface area, and volume. Notably, the calcium carbonate coating formed by microbial mineralization on the particle surface significantly reduced the overall flatness of the particles and somewhat increased their sphericity. This provides an intuitive understanding of the changes in particle size and morphology due to MICP treatment.
2. The calcium carbonate coating formed by microbial mineralization on the particle surface significantly increased the coating thickness. SEM results indicated that the surfaces of MICP-treated, coated calcareous sand particles became smoother, with the original unevenness reduced and surface pores filled with calcium carbonate, all contributing to enhanced mechanical properties.
3. The concentration of the cementation solution can regulate the thickness of the calcium carbonate coating, directly influencing the crushing behavior. At a cementation solution concentration of 0.5 mol/L, the calcium carbonate coating was thinner, resulting in a lower particle crushing strength. When increased to 1 mol/L, the coating thickness and crushing strength were maximized, with some particles exhibiting crushing strengths exceeding 40 MPa, demonstrating optimal microbial mineralization effects. Further increasing the concentration to 1.5 mol/L inhibited microbial activity due to the excessively high concentration, reducing coating thickness and crushing strength, but most particles still maintained relatively high crushing strength. These findings highlight the crucial role of controlling the cementation solution concentration in optimizing the mechanical properties of calcareous sand. Specifically, at 1 mol/L, the thickening of the coating significantly enhanced the particles’ crushing strength and energy, whereas at 1.5 mol/L, despite a decrease in crushing energy, the initial larger particle size maintained high crushing energy levels. Additionally, with increasing coating thickness, the Weibull modulus decreased, reflecting increased dispersion in the crushing strength, further proving the direct and significant impact of calcium carbonate coating thickness on the crushing characteristics of the particles.
4. In compression experiments, coated calcareous sand particles exhibited different crushing modes, including explosive crushing, mixed crushing, and splitting crushing, closely related to the presence of the calcium carbonate coating. As compression increased, particles first showed elastic deformation, quickly reaching peak stress and fracturing. The filling effect of the calcium carbonate enhanced the overall crushing strength of the particles, causing them to tend to break into larger fragments, especially when the coating was thicker. This phenomenon underscores that the MICP treatment, by altering the coating thickness, not only improved the mechanical properties of calcareous sand but also effectively controlled the crushing behavior of the particles.
5. During single-particle crushing, the fractal characteristics of coated calcareous sand particles were expressed by the relationship between the number of fragments and particle size, following a fractal distribution. Changes in the fractal dimension indicated that with increasing coating thickness, the fractal degree of local crushing decreased, related to the increased thickness of the calcium carbonate coating formed after the MICP treatment. This variation in coating thickness had a direct impact on the local crushing characteristics of the particles.

## Figures and Tables

**Table 1:** The composition and quantities of the chosen culture medium.

Components	Dosage
Yeast extract	20 g/L
NH_4_Cl	20 g/L
MnSO_4_·H_2_O	12 mg/L
NiCl_2_·6H_2_O	24 mg/L
Agar	15 g/L (Solid medium)
NaOH or HCl (pH control)	pH: 8.5~9.0

**Table 2:** CS concentration, coating thickness, characteristic stress, and Weibull modulus of test particles.

Test Group	Coating Thickness (mm)	Characteristic Stress (MPa)	Weibull Modulus (m)
α_1_	0	21.607	0.13912
α_2_	0.219	26.568	0.12607
α_3_	0.369	34.402	0.09775
α_4_	0.361	29.357	0.12304

**Table 3:** Single-particle energy distribution of calcareous sand and coated calcareous sand.

Test Group	Coating Thickness (mm)	Test Particles Crushing Force (N)	Test Particles Crushing Energy (mJ)
α_1_	0	236.3	473.2
α_2_	0.219	314.4	628.8
α_3_	0.369	330.6	826.5
α_4_	0.361	354.1	1416.4

**Table 4:** Coating thickness, Weibull modulus, and fractal dimension of test particles.

Test Number	Coating Thickness (mm)	Weibull Modulus (m)	Fractal Dimension
α_1_	0	0.13912	0.366
α_2_	0.219	0.12607	0.336
α_3_	0.369	0.09775	0.267
α_4_	0.361	0.12304	0.329

## Data Availability

Data will be made available on request.
